# The Protective Effect of Aspirin Eugenol Ester on Oxidative Stress to PC12 Cells Stimulated with H_2_O_2_ through Regulating PI3K/Akt Signal Pathway

**DOI:** 10.1155/2021/5527475

**Published:** 2021-06-24

**Authors:** Zhen-Dong Zhang, Ya-Jun Yang, Xi-Wang Liu, Zhe Qin, Shi-Hong Li, Li-Xia Bai, Jian-Yong Li

**Affiliations:** Key Laboratory of New Animal Drug Project of Gansu Province, Key Laboratory of Veterinary Pharmaceutical Development of Ministry of Agriculture and Rural Affairs, Lanzhou Institute of Husbandry and Pharmaceutical Sciences of CAAS, Lanzhou 730050, China

## Abstract

Aspirin eugenol ester (AEE) is a new pharmaceutical compound esterified by aspirin and eugenol, which has anti-inflammatory, antioxidant, and other pharmacological activities. This study is aimed at identifying the protective effect of AEE against H_2_O_2_-induced apoptosis in rat adrenal pheochromocytoma PC12 cells and the possible mechanisms. The results of cell viability assay showed that AEE could increase the viability of PC12 cells stimulated by H_2_O_2_, while AEE alone had no significant effect on the viability of PC12 cells. Compared with the control group, the activities of superoxide dismutase (SOD), catalase (CAT), and glutathione peroxidase (GSH-Px) were significantly decreased, and the content of malondialdehyde (MDA) was significantly increased in the H_2_O_2_ group. By AEE pretreatment, the level of MDA was reduced and the levels of SOD, CAT, and GSH-Px were increased in H_2_O_2_-stimulated PC12 cells. In addition, AEE could reduce the apoptosis of PC12 cells induced by H_2_O_2_ via reducing superoxide anion, intracellular ROS, and mitochondrial ROS (mtROS) and increasing the levels of mitochondrial membrane potential (*ΔΨ*m). Furthermore, the results of western blotting showed that compared with the control group, the expression of p-PI3K, p-Akt, and Bcl-2 was significantly decreased, while the expression of Caspase-3 and Bax was significantly increased in the H_2_O_2_ group. In the AEE group, AEE pretreatment could upregulate the expression of p-PI3K, p-Akt, and Bcl-2 and downregulate the expression of Caspase-3 and Bax in PC12 cells stimulated with H_2_O_2_. The silencing of PI3K with shRNA and its inhibitor-LY294002 could abrogate the protective effect of AEE in PC12 cells. Therefore, AEE has a protective effect on H_2_O_2_-induced PC12 cells by regulating the PI3K/Akt signal pathway to inhibit oxidative stress.

## 1. Introduction

There are growing evidences that oxidative stress is closely related to human neurodegenerative diseases, including Alzheimer's disease (AD) and Huntington's disease (HD) [1–5]. Excessive production of reactive oxygen species (ROS) is one of the main causes of oxidative stress [6–9]. ROS in the body mainly includes hydroxyl radicals, superoxide anions, and singlet oxygen [10–12]. The normal level of ROS helps to maintain normal cell function. However, excessive ROS stimulates cells not only to cause structural damage and promotes oxidative stress but also destroy the redox balance and lead to cell damage and apoptosis [13–15]. There are many closely related antioxidant systems in the body. The main role of antioxidant systems is to prevent oxidative damage to the body by removing excess ROS from cells [16–18]. In fact, the dynamic balance of oxidants and antioxidants in the body is very important for neuroprotection [19–21]. The key antioxidant enzymes in cells are SOD, CAT, and GSH-Px [22–24]. ROS-mediated oxidative stress mainly activates the inherent apoptosis pathway by releasing a variety of prodeath factors into the cytoplasm of damaged mitochondria [25–27]. Among them, the PI3K/Akt pathway is an important signal pathway to promote neuronal survival. Studies have shown that it can affect cell survival by inhibiting the expression of proapoptotic protein and inducing the expression of antiapoptotic protein of Bcl-2 family [28–30].

As a new compound, AEE plays an active role in many aspects [31–40]. AEE can prevent tail thrombosis induced by kappa-carrageenan in rats [39]. At the same time, AEE can attenuate thrombus induced with high-fat diet in rats by regulating hemorheology and blood biochemistry [37]. With a further study, a rat model of blood stasis was established and it was observed that AEE could alleviate the symptoms of blood stasis in rats [41]. It was also found that AEE can inhibit agonist-induced platelet aggregation in rats by regulating PI3K/Akt signal pathways [33]. AEE has not only the effects of anti-inflammation, antithrombosis, and antiblood stasis but also the effect of antiatherosclerosis and other cardiovascular diseases. The previous studies proved that AEE had an antioxidant effect and could reduce H_2_O_2_-induced mitochondrial dysfunction by regulating Bcl-2 and Nrf2 [32, 34]. It is not clear whether AEE can play a neuroprotective role in neurodegenerative diseases. The purpose of this study was to explore whether AEE can attenuate H_2_O_2_-induced oxidative damage in PC12 cells and its possible mechanism.

## 2. Materials and Methods

### 2.1. Chemicals

3% H_2_O_2_ solution and dimethyl sulfoxide were obtained from Sigma (St. Louis, MO). RPMI-1640 culture medium, 0.05%Trypsin-EDTA, and fetal bovine serum (FBS) were from Gibco (Grand Island, NY, USA). One step TUNEL apoptosis assay kit, puromycin dihydrochloride, bicinchoninic acid assay kit, glutathione peroxidase kit, catalase assay kit, DAPI staining solution, DAF-FM diacetate kit, dihydroethidium, superoxide dismutase, and malondialdehyde assay kit were obtained from Beyotime (Shanghai, China). Anti-Bax, anti-Bcl-2, and anti-Caspase-3 were from Abcam (Cambridge, MA, USA). Anti-PI3K, anti-Akt, antiphosphorylation-PI3K, and antiphosphorylation-Akt were purchased from Cell Signaling Technology, Inc. (Beverly, MA, USA). Immobilon-PSQ transfer membrane was obtained from Millipore (Billerica, MA, USA). An Annexin V/FITC apoptosis detection kit was from BD Biosciences (San Diego, CA, USA). PI3-kinase LY 294002 was purchased from MedChemExpress LLC (New Jersey, USA). Lipofectamine™ 3000 transfection reagent was purchased from Thermo Fisher Scientific, Inc. (Invitrogen, USA). Lentivirus control and PI3K shRNA (U6-MCS-Ubiquitin Cherry-IRES-puromycin) were purchased from GeneChem (Shanghai, China).

### 2.2. Cell Cultures and Cell Treatment

PC12 cells were routinely maintained in RPMI-1640 medium containing 10% FBS (*v*/*v*) at 37°C in a humidified atmosphere of 5% CO_2_ and then randomly divided into the control group, H_2_O_2_ group, and AEE pretreatment group.

### 2.3. Cell Viability

Cell viability was determined via CCK-8 assay. Briefly, PC12 cells (1 × 10^4^ cells/well) were plated on a 96-well culture plate and incubated for 24 h. 10 *μ*L CCK-8 solution was added to each well. The number of viable cells was assessed by the measurement of the absorbance at 450 nm.

### 2.4. TUNEL Staining

PC12 cells (5 × 10^4^ cells/well) were seeded into 12-well culture plates. After treatment, cells were washed with PBS and fixed with 4% paraformaldehyde in PBS at 25°C for 30 min. After the cells were washed with PBS twice, 0.3% Triton X-100 PBS was added and incubated at 25°C for 5 min. The wells were washed twice with PBS, and TUNEL detection solution was added. After incubation of cells at 37°C for 1 h, DPAI staining solution was added and incubated at room temperature for 20 min. The cells were washed with PBS. Images were captured using a scanning laser confocal microscope (LSM800, Carl Zeiss, Germany).

### 2.5. Flow Cytometric Analysis

PC12 cells were seeded into a 6-well plate. After treatments, PC12 cells were assessed using the corresponding commercial kit according to the manufacturer's protocols [34]. PC12 cells were sorted by a flow cytometer (BD FACSVerse, CA, USA), and the data were analyzed with FlowJo 7.6.

### 2.6. Mitochondrial Membrane Potential (*ΔΨ*m) Assays

The *ΔΨ*m was determined using MitoTracker® Red CMXROS (Invitrogen; Thermo Fisher Scientific, Inc.). Briefly, the cells were seeded in 12-well plates. MitoTracker® Red probe was directly added into the culture media and incubated for 30 min at 37°C in the dark. Images were captured using a scanning laser confocal microscope (LSM800; Carl Zeiss, Germany).

### 2.7. Measurement of Intracellular Superoxide Anion and Total Intracellular and Mitochondrial ROS Generation

Intracellular and mitochondrial ROS generation and superoxide anion were measured using a DCFH-DA or MitoSOX™ red probe or Dihydroethidium (DHE) as previously described [42].

### 2.8. Determination of MDA, SOD, GSH-Px, and CAT

The activities of MDA, SOD, GSH-Px, and CAT in PC12 cells were assessed using the corresponding commercial kits according to the manufacturer's protocols [43, 44].

### 2.9. Protein Expression Analysis

The expression of Bcl-2, Bax, Caspase-3, PI3K, Akt, phospho-PI3K, and phospho-Akt was assessed by western blot analysis. Cell samples were lysed on ice with lysis buffer containing cocktail proteinase inhibitors and protein phosphatase inhibitors (Thermo Fisher Scientific, Inc., Rockford, USA). The protein concentration was quantified using a bicinchoninic acid (BCA) assay kit (Beyotime, Shanghai, China). Protein samples were separated by SDS-PAGE using 4-20% precast gradient polyacrylamide gels (Shanghai Suolaibao Bio-Technology Co., Ltd., Shanghai, China). After separation by SDS-PAGE, proteins were transferred to a PVDF membrane. The blots were then incubated with primary antibodies and subsequently incubated with horseradish peroxidase- (HRP-) conjugated secondary antibodies. The results were detected using G:Box Chemi XRQ imaging system (Cambridge, Britain).

### 2.10. Cell Transfection

Lentiviral vectors expressing PI3K shRNA or control shRNA were obtained from GeneChem (Shanghai, China). Following the manufacturer's protocol, PC12 cells were cotransfected with lentivirus and packaging vectors using Lipofectamine 3000. Lentiviruses were harvested 48 h after transfection, centrifuged, and filtered through 0.45 *μ*m membrane filters (Millipore). Lentiviruses were transduced in 50% confluent PC12 cells. Stable cells were selected by selected in 1 *μ*g/mL puromycin.

### 2.11. Determination of Apoptosis after Inhibition of Signal Pathway

The PI3K/Akt signaling pathways in the PC12 cells were inhibited by short hairpin RNA (shRNA) and inhibitor LY 294002 against PI3K. In this part, it was divided into eleven groups. These PC12 cells were treated with 4.0 *μ*M AEE and H_2_O_2_ according to the protocol described in Section 2.2.

### 2.12. Statistical Analysis

The statistical analysis was performed using SAS 9.2 software (SAS Institute Inc., Cary, NC, USA). Statistical significance was defined as *P* value < 0.05. The statistical analyses were applied to selected pairs.

## 3. Results

### 3.1. AEE Protects the Cell Viability of H_2_O_2_-Stimulated PC12 Cells

**Figure 1:**
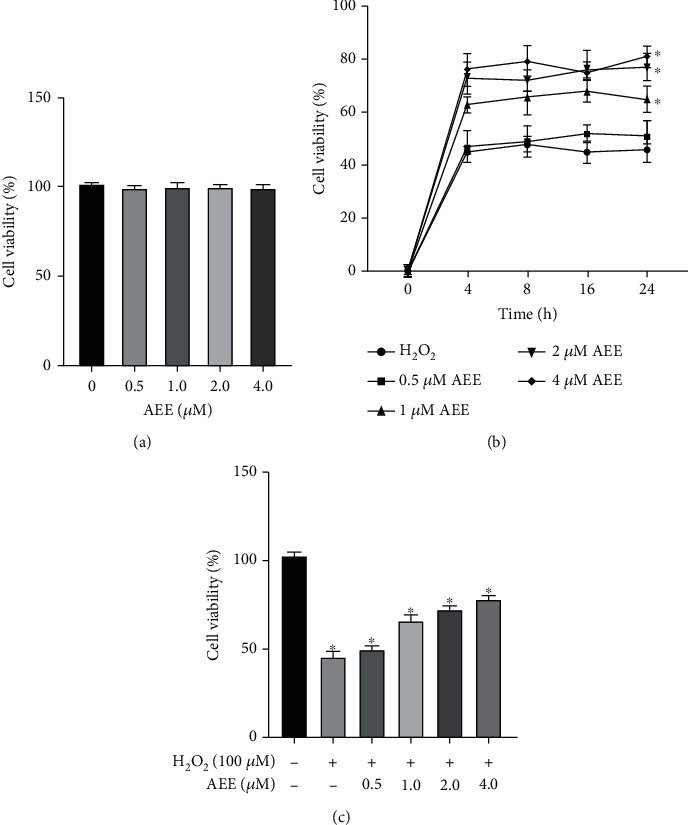
AEE pretreatment enhances the viability of PC12 cells induced by H_2_O_2_. (a) Effects of different concentrations of AEE on the viability of PC12 cells. (b) Effects of AEE at different concentrations and at different times on H_2_O_2_-induced viability of PC12 cells. (c) Effects of pretreatment with different concentrations of AEE on the viability of PC12 cells induced by H_2_O_2_. ^∗^*P* < 0.05 compared with control group; ^#^*P* < 0.05 compared with the H_2_O_2_ group. “+”: with the treatments in the PC12 cells; “−”: without the treatments in the PC12 cells.

As shown in Figure 1(a), the different concentrations of AEE had no significant effect on PC12 cell viability. Compared with the control group, 100 *μ*M H_2_O_2_ treatment for 12 h could significantly decrease the viability of PC12 cells (Figures 1(b) and 1(c)). As shown in Figure 1(b), the optimal working time for AEE was 8 h. Compared with the H_2_O_2_ group, the viability of PC12 cells increased significantly after being pretreated with different concentrations of AEE (1.0, 2.0, and 4.0 *μ*M) for 8 h (Figures 1(b) and 1(c)).

### 3.2. AEE Inhibits H_2_O_2_-Induced Apoptosis in PC12 Cells

**Figure 2:**
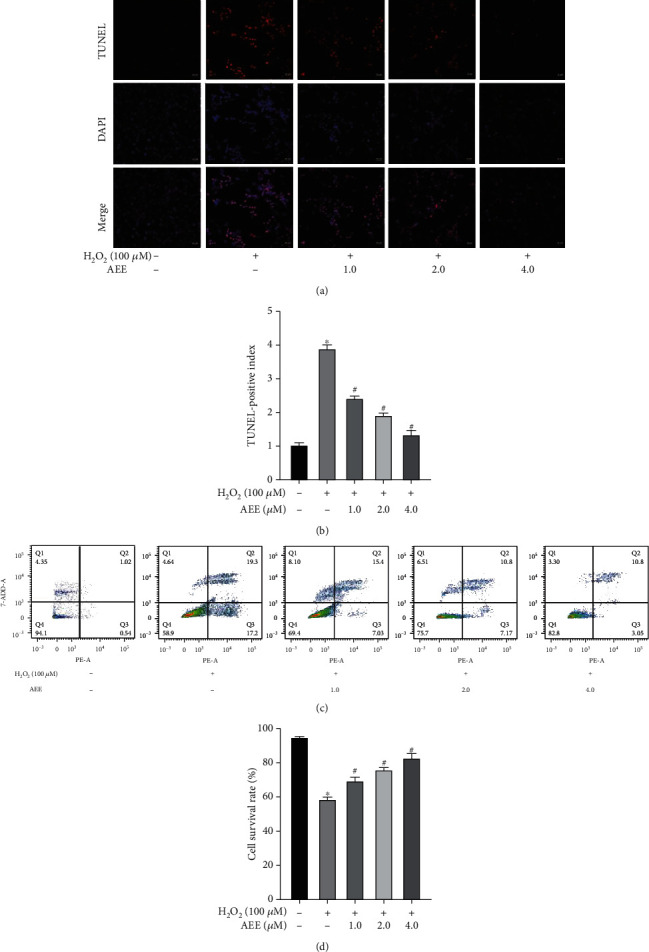
AEE pretreatment attenuates H_2_O_2_-induced apoptosis in PC12 cells. (a, b) Apoptosis was detected by DAPI and TUNEL staining. Scale bar = 20 nm. (c, d) Apoptotic assay by flow cytometry. ^∗^*P* < 0.05 compared with the control group; ^#^*P* < 0.05 compared with the H_2_O_2_ group. “+”: with the treatments in the PC12 cells; “−”: without the treatments in the PC12 cells.

The apoptosis morphology was evaluated by DAPI staining. Compared with the control group, the nucleus was significantly smaller, the brightness was enhanced, and there were characteristics of apoptosis in the H_2_O_2_ group (Figure 2(a)). To assess apoptosis-induced DNA fragmentation, TUNEL assay was performed. Compared with the control group, the number of TUNEL-positive cells (red fluorescence) of PC12 cells treated with H_2_O_2_ increased significantly (Figures 2(a) and 2(b)). The results of flow cytometry showed that H_2_O_2_ can increase significantly PC12 cell apoptosis, while AEE can inhibit the H_2_O_2_-induced PC12 cell apoptosis (Figures 2(c) and 2(d)). Moreover, the different concentrations of AEE (1.0, 2.0, and 4.0 *μ*M) can inhibit the apoptosis of PC12 cells induced by H_2_O_2_.

### 3.3. AEE Antagonizes H_2_O_2_-Induced Oxidative Stress in PC12 Cells

**Figure 3:**
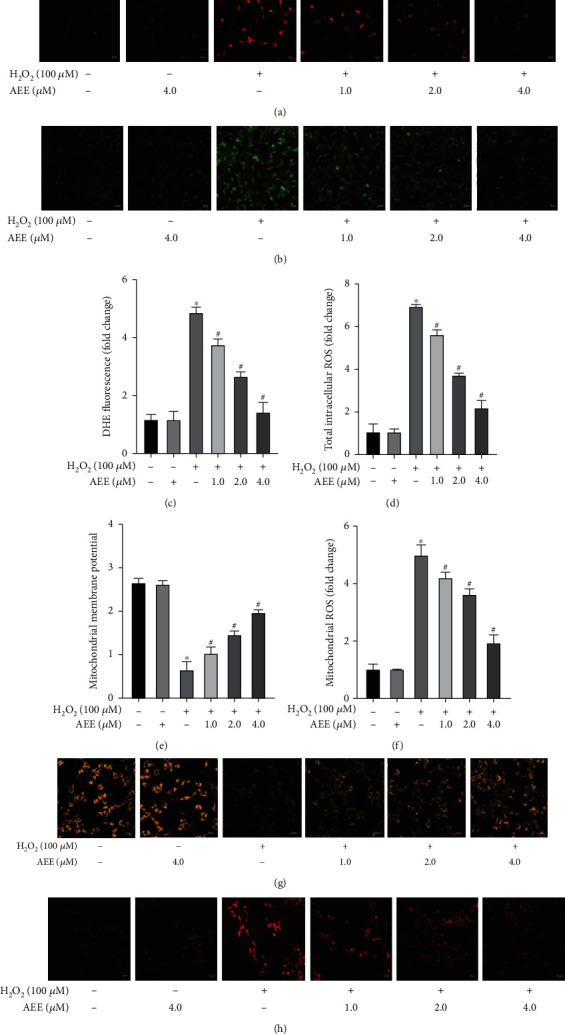
AEE antagonizes H_2_O_2_-induced oxidative stress in PC12 cells. (a, c) The representative images from DHE staining and fluorescence intensity of DHE. (b, d) The representative images from DCFH-DA staining and fluorescence intensity of DCF. (e, g) The representative images from *ΔΨ*m staining and fluorescence intensity of *ΔΨ*m. (f, h) The representative images from mtROS staining and fluorescence intensity of mtROS. ^∗^*P* < 0.05, compared with the control group; ^#^*P* < 0.05, compared with the H_2_O_2_ group. “+”: with the treatments in the PC12 cells; “−”: without the treatments in the PC12 cells.

To verify the changes of the redox state of PC12 cells, the levels of superoxide anion, total reactive oxygen species, and mitochondrial reactive oxygen species (mtROS) were detected. AEE alone did not change the levels of superoxide anion, intracellular ROS, and mtROS. Compared with the control group, 100 *μ*M H_2_O_2_ could significantly increase the levels of superoxide anion, intracellular total ROS, and mtROS. Pretreatment with 1.0, 2.0, and 4.0 *μ*M AEE for 8 h could significantly inhibit the production of superoxide anion, total ROS, and mtROS induced by H_2_O_2_ (Figures 3(a)–3(d), 3(f), and 3(h)). In addition, the mitochondrial membrane potential (*ΔΨ*m) was further detected. The results showed that AEE alone did not affect the *ΔΨ*m of PC12 cells (Figures 3(e) and 3(g)). AEE pretreatment could significantly increase *ΔΨ*m in PC12 cells, compared with the H_2_O_2_ group (Figures 3(e) and 3(g)). The results showed that AEE could significantly alleviate the mitochondrial dysfunction of PC12 cells via inhibiting intracellular ROS, mtROS, and superoxide anion levels.

### 3.4. AEE Enhances the Enzymatic Activities of ROS-Scavenging Enzymes in H_2_O_2_-Stimulated PC12 Cells

**Figure 4:**
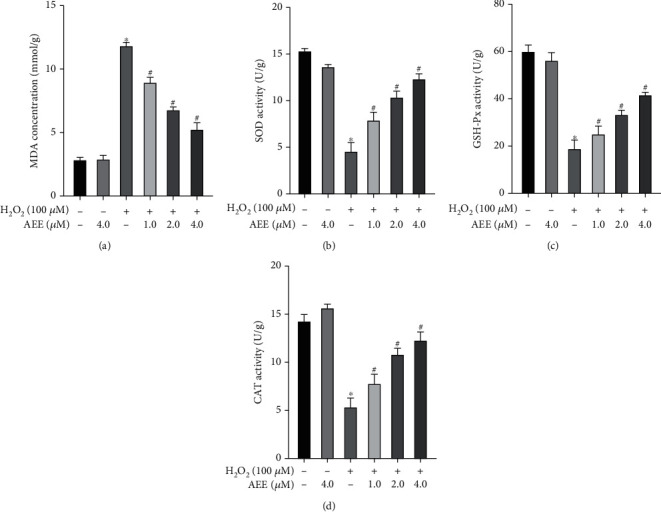
AEE increases the activity of scavenging ROS enzyme in PC12 cells induced by H_2_O_2_. (a) The level of MDA was measured. (b) The activity of SOD was measured. (c) The activity of GSH-Px was measured. (d) The activity of CAT was measured. ^∗^*P* < 0.05, compared with the control group; ^#^*P* < 0.05, compared with H_2_O_2_ group. “+”: with the treatments in the PC12 cells; “−”: without the treatments in the PC12 cells.

The activities of MDA, SOD, GSH-Px, and CAT in cells were detected to explore the possible mechanism of AEE attenuating H_2_O_2_-induced injury in PC12 cells. H_2_O_2_ could significantly increase the activity of MDA and decrease the activity of SOD, GSH-Px, and CAT, compared with the control group. However, AEE pretreatment could significantly increase the activities of SOD, GSH-Px, and CAT and decrease the activity of MDA (Figures 4(a)–4(d)). These results suggest that AEE pretreatment may attenuate H_2_O_2_-induced oxidative damage in PC12 cells by increasing the enzymatic activities of ROS-scavenging enzymes.

### 3.5. AEE Regulates the Expression of Apoptosis-Related Proteins in H_2_O_2_-Stimulated PC12 Cells

**Figure 5:**
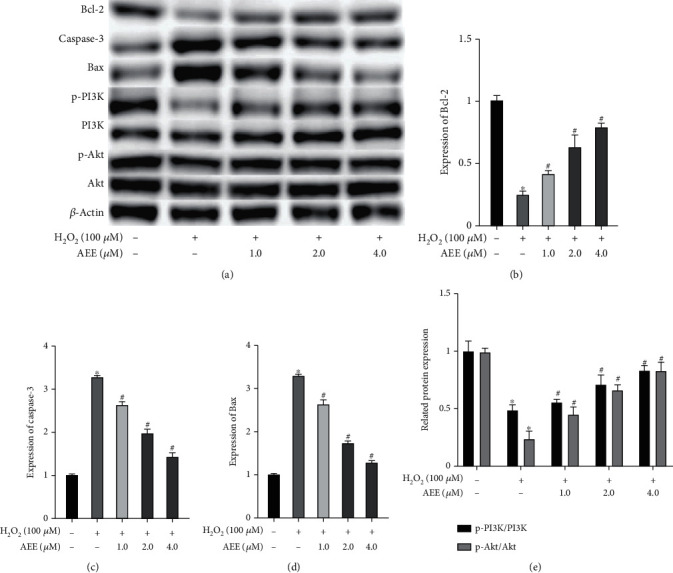
AEE regulates the expression of the related proteins in PC12 cells induced by H_2_O_2_. (a–d) The expression of Bcl-2, Bax, and Caspase-3 was measured. (a, e) The expression of p-PI3K, PI3K, p-Akt, and Akt was measured. ^∗^*P* < 0.05, compared with the control group; ^#^*P* < 0.05, compared with the H_2_O_2_ group. “+”: with the treatments in the PC12 cells; “−”: without the treatments in the PC12 cells.

To further explore the molecular mechanism of AEE attenuating H_2_O_2_-induced apoptosis in PC12 cells, we used western blotting to detect the expression of Caspase-3, Bcl-2, and Bax. As shown in Figures 5(a) and 5(b), compared with the control group, H_2_O_2_ could significantly increase the expression of Bax and Caspase-3 and decrease the expression of Bcl-2. Compared with the H_2_O_2_ group, AEE could significantly reverse the above changes.

### 3.6. Effect of AEE on the PI3K/Akt Signal Pathway in PC12 Cells Stimulated by H_2_O_2_

The PI3K/Akt signaling pathway plays an important role in regulating neuronal apoptosis [45, 46]. As shown in Figure 5, H_2_O_2_ significantly decreased the expression of p-Akt and p-PI3K but had no significant effect on the expression of Akt and PI3K, compared with the control group. Compared with the H_2_O_2_ group, AEE pretreatment could significantly upregulate the expression of p-Akt and p-PI3K in PC12 cells (Figures 5(a) and 5(b)). Interestingly, AEE also had no significant effect on the expression of Akt and PI3K in PC12 cells. These results showed that AEE may have a protective effect on H_2_O_2_-induced PC12 cells via the PI3K/Akt pathway.

**Figure 6:**
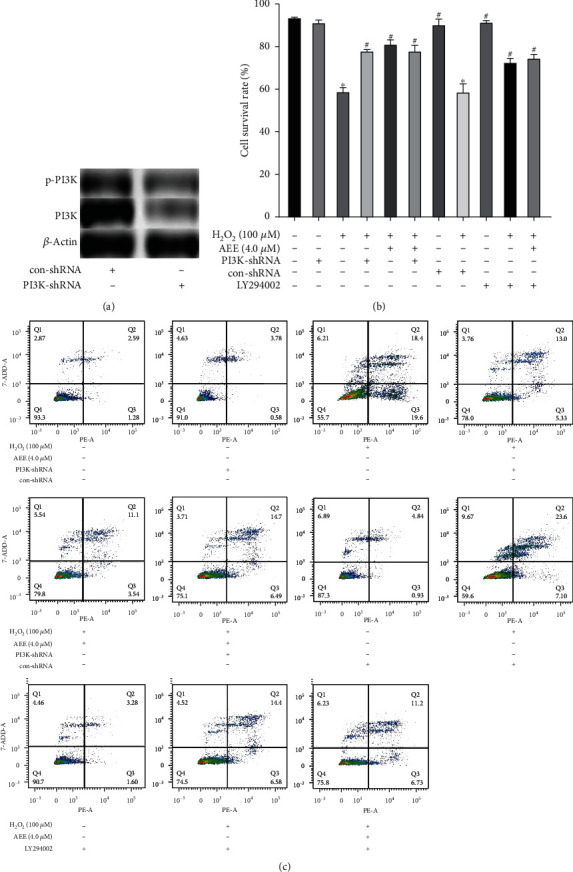
Intervention of PI3K with an inhibitor and shRNA reduced the effect of AEE on H_2_O_2_-induced apoptosis. (a) The expression of p-PI3K and PI3K in the control-shRNA treatment groups and PI3K-shRNA treatment groups. (b, c) Apoptotic assay by flow cytometry. ^∗^*P* < 0.05, compared with the control group; ^#^*P* < 0.05, compared with the H_2_O_2_ group.

PI3K inhibitors LY294002 and shRNA were used to inhibit the expression of PI3K (Figure 6(a)). The results of flow cytometry showed that compared with the control group, the apoptosis rate of PC12 cells in the H_2_O_2_ group increased significantly, while the AEE+LY294002 treatment group and AEE+PI3K shRNA group could not inhibit the apoptosis of PC12 cells induced by H_2_O_2_ (Figures 6(b) and 6(c)). The above results suggested that AEE can alleviate the oxidative damage of PC12 cells induced by H_2_O_2_ via the PI3K/Akt pathway.

## 4. Discussion

The above studies suggested that AEE attenuates H_2_O_2_-induced oxidative damage in PC12 cells via inhibiting oxidative stress. It is mainly manifested in inhibiting the production of superoxide anion, MDA, intracellular ROS, and mtROS and increasing the activity of CAT, SOD, and GSH-Px.

PC12 cell line is derived from rat pheochromocytoma [18, 47, 48]. Because of the high permeability of the plasma membrane to H_2_O_2_, H_2_O_2_-induced PC12 cells are generally considered to be an ideal cell model for studying neurodegenerative diseases [49–51]. Studies showed that the imbalance between free radical accumulation and antioxidant defense seems to be a link between cell death and the progression of neurodegenerative diseases [1, 9, 52]. ROS and the resulting oxidative stresses play an important role in apoptosis. H_2_O_2_ is an important source of intracellular ROS, because it can penetrate the cell membrane and can be converted into other free radicals, such as superoxide anions and hydroxyl radicals [53, 54]. H_2_O_2_ can also cause serious damage to cells by attacking biomolecule membranes and eventually lead to apoptosis [55, 56]. The results of cell viability showed that the viability of PC12 cells decreased with approximately 50% after 12 h of 100 *μ*M H_2_O_2_ stimulation. AEE pretreatment could significantly increase the viability of PC12 cells induced by H_2_O_2_.

Excessive ROS can lead to cell dysfunction and apoptosis, especially in neurodegenerative diseases [57–59]. Previous studies found that H_2_O_2_ could cause excessive accumulation of intracellular ROS, mtROS, and superoxide anion in PC12 cells. AEE pretreatment could reduce the increase of intracellular ROS, mtROS, and DHE in PC12 cells induced by H_2_O_2_. MDA can cause damage to the cell membrane [60–62]. It is also an important biomarker to evaluate the level of oxidative stress in cells [61, 63, 64]. In addition, there are a variety of scavenging active oxygen enzymes in organisms, such as SOD, CAT, and GSH-Px [65–67]. Under normal physiological conditions, these antioxidant enzymes work together to maintain the redox balance of the body [68]. SOD catalyzes the conversion of superoxide radicals to O_2_ and H_2_O_2_, while CAT catalyzes dismutation reactions of H_2_O_2_ into H_2_O [69, 70]. GSH-PX prevents the formation of toxic hydroxyl and peroxyl radicals via providing electrons to H_2_O_2_ and lipid peroxides [71]. Studies showed that H_2_O_2_ induced PC12 cells could produce excessive MDA, intracellular ROS, and mtROS and significantly reduce the activities of SOD, GSH-Px, and CAT. AEE pretreatment not only decreased the levels of MDA, intracellular ROS, and mtROS of PC12 cells but also increased the activities of SOD, GSH-Px, and CAT. As previously reported, the accumulation of ROS can lead to mitochondrial dysfunction by depolarizing mitochondrial membrane potential [72, 73]. Mitochondrial membrane potential (*ΔΨ*m) is a sensitive index to measure the function of mitochondria [74, 75]. The results showed that there was an obvious apoptosis in PC12 cells after H_2_O_2_ stimulation, and the cell viability and *ΔΨ*m decreased. As expected, AEE pretreatment could reduce H_2_O_2_-induced apoptosis. These results suggested that AEE may reduce the apoptosis of PC12 cells induced by H_2_O_2_ via inhibiting the excessive production of ROS.

**Figure 7:**
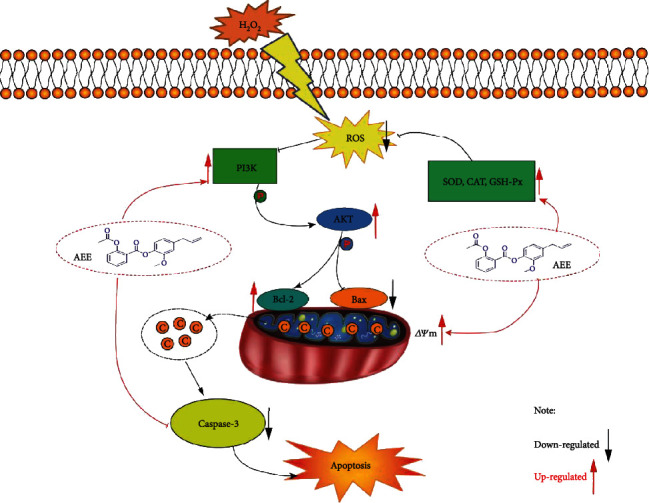
The molecular mechanism of AEE inhibiting H_2_O_2_-induced apoptosis in PC12 cells.

The PI3K/Akt signaling pathway plays an important role in cell survival, differentiation, proliferation, and apoptosis [76–78]. Phosphatidylinositol 3 kinase (PI3Ks) belongs to the lipid kinase family, which phosphorylates inositol phosphate at the D-3 position of the inositol head group, resulting in the production of the D-3 phosphate. PI3K mediates extracellular signal transduction and regulates a variety of cellular events, including cell mitosis, cell survival, and membrane transport. According to the enzyme domain structure and substrate specificity of PI3K, it can be divided into three categories in mammals (I-III). Among them, the class I subfamily is the most widely studied. The class I subfamily consists of four catalytic subunits, including three IA subunits (p110 *α*, p110 *β*, and p110 *δ*) and one IB subunit (p110 *γ*). When phosphorylation of PI3K increases, it transduces signals through inositol 3-phosphate-dependent protein kinase-1 (PDK1), a serine/threonine kinase. PDK1 is recruited to the cell membrane after PI3K activation, where it phosphorylates and activates Akt, the main medium of the PI3K signal transduction pathway. Akt, a serine/threonine kinase, is pivotal in cellular metabolism, growth, and survival [79, 80]. When Akt is activated, it plays a key role in PI3K-mediated signal transduction [81–83]. The phosphorylation of AKT can increase the expression of Bcl-2 and inhibit the expression of Bax in mitochondria. LY294002 is not only a competitive DNA-PK inhibitor but also a commonly used PI3K drug inhibitor, which acts on the ATP binding site of PI3K enzyme, thus selectively inhibiting PI3K-Akt connection. Pretreatment with LY294002 for 2 h significantly counteracted the protective effect of AEE. Consistent with this, using shRNA to knock down PI3K has a similar result. H_2_O_2_ treatment of PC12 cells resulted in excessive production of intracellular ROS. The phosphorylation of PI3K can be inhibited by excessive production of ROS. However, AEE pretreatment could inhibit the decrease of PI3K phosphorylation induced by H_2_O_2_. With the recovery of mitochondrial membrane potential, mitochondria will reduce the release of cytochrome c and inhibit the activation of caspase family. At the same time, the enzyme activity of CAT, SOD, and GSH-Px was changed by AEE pretreatment, which further eliminated the excess ROS in the PC12 cells (Figure 7). The results showed that AEE can alleviate H_2_O_2_-induced apoptosis of PC12 cells via upregulating the expression of p-PI3K, p-Akt, and Bcl-2 and downregulating the expression of Caspase-3 and Bax.

## 5. Conclusion

AEE may inhibit oxidative stress by regulating the PI3K/Akt signal pathway, thus protecting PC12 cells from apoptosis induced by H_2_O_2_. It is suggested that AEE may be a new potential drug to treat neurodegenerative diseases caused by oxidative stress.

## Data Availability

The data used to support the findings of this study are included within the article.
